# Clinical Comparation of Extra-Short (4 mm) and Long (>8 mm) Dental Implants Placed in Mandibular Bone: A Systematic Review and Metanalysis

**DOI:** 10.3390/healthcare9030315

**Published:** 2021-03-12

**Authors:** Vittorio Moraschini, Carlos Fernando de Almeida Barros Mourão, Pietro Montemezzi, Ingrid Chaves Cavalcante Kischinhevsky, Daniel Costa Ferreira de Almeida, Kayvon Javid, Jamil Awad Shibli, José Mauro Granjeiro, Monica Diuana Calasans-Maia

**Affiliations:** 1 Periodontology Department, Dental Research Division, School of Dentistry, Veiga de Almeida University, Rio de Janeiro 20271-020, Brazil; vitt.mf@gmail.com; 2 Biotechnology Department, Universidade Federal Fluminense, Niteroi 24020-140, Brazil; 3 Private Practice, 24128 Bergamo, Italy; m.montemezzi@libero.it; 4 Graduate Program, Dentistry School, Universidade Federal Fluminense, Niteroi 24020-140, Brazil; ingrid.chaves@hotmail.com (I.C.C.K.); drdanieldealmeida@gmail.com (D.C.F.d.A.); onecure@aol.com (K.J.); 5 Periodontology and Oral Implantology Department, University of Guarulhos, Guarulhos 07023-070, Brazil; jashibli@yahoo.com; 6 Bioengineering Laboratory, National Institute of Metrology, Quality and Technology (INMETRO), Duque de Caxias 25250-020, Brazil; jmgranjeiro@gmail.com; 7 Dental School, Fluminense Federal University, Niterói 24020-140, Brazil; 8 Oral Surgery Department, Universidade Federal Fluminense, Niteroi 24020-140, Brazil; monicacalasansmaia@gmail.com

**Keywords:** dental implants, short dental implants, marginal bone loss, systematic review

## Abstract

This systematic review (SR) aimed to evaluate implant survival rate, marginal bone loss (MBL), and biological/prosthetic complications of extra-short 4 mm dental implants. An electronic search without language or date restrictions was performed in five databases and in gray literature for articles published until August 2020. Prospective cohort studies and randomized clinical trials (RCTs) that evaluated the clinical performance of extra-short 4 mm dental implants were included. Studies were independently assessed for risk of bias using the Cochrane Collaboration’s tool. The protocol of this SR was registered in the PROSPERO database under number CRD42019139709. Four studies were included in the present SR. There was no significant difference in implant survival rate (*p* = 0.75) between extra-short 4 mm and long implants. After 12 months of function, the extra-short implants had a significantly (*p* = 0.003) lower marginal bone loss (MBL) rate when compared to long implants. Extra-short implants had a lower number of biological and prosthetic complications when compared to long implants. After 12 months of follow-up, extra-short 4 mm dental implants placed in the mandible exhibit satisfactory clinical outcomes concerning implant survival rate and MBL when compared to longer implants, with a low number of biological and prosthetic complications. A higher number of RCTs with longer follow-up is necessary for the future.

## 1. Introduction

Osseointegration has proved to be predictable in implant dentistry, but some clinical challenges demand treatment alternatives with predictability and low morbidity [1,2,3,4]. The resorption of bone tissue after teeth extraction could represent a great challenge for oral rehabilitation with dental implants [5,6]. The literature reports that bone regeneration with autogenous onlay grafts, guided bone regeneration, and bone distraction are viable options to treat bone resorption [7,8,9,10,11]. However, such techniques have associated disadvantages such as morbidity, the need for multiple procedures, a high cost, patient acceptance, and a significant incidence of postoperative complications [12,13,14,15,16,17]. For these reasons, contemporaneously, there is a tendency for an increase in the demand for minimally invasive procedures.

The definition of a short dental implant is controversial; the most accepted definition is that it comprises implants <10 mm [18,19], and an extra-short implant should measure ≤6 mm [19,20]. However, other authors consider that extra-short implants must have a length of 5 mm or less [21]. Currently, these two modalities of treatment in regions with deficiency of bone height in the jaws present high rates of survival and success [19,22]. A recent systematic review (SR) evaluated the effectiveness of extra-short implants (5 and 6 mm in length). They concluded that this alternative is feasible in ridges exhibiting atrophy, demonstrating a satisfactory survival rate, as well as a low rate of prosthetic and biologic complications across to a five-year follow-up [23].

Currently, extra-short implants 4 mm in length are commercially available for the rehabilitation of cases with extreme bone atrophy. Although some RCTs [22,24] have evaluated the clinical performance of these implants, there is still no consensus in the literature about long-term performance. Thus, this SR aimed to compare the survival rate, marginal bone loss (MBL), as well as biological and prosthetic complications of 4 mm splinted implants to >8 mm implants in a mandible.

## 2. Materials and Methods

### 2.1. Protocol and Registration

The protocol for this SR was based on PRISMA-P [25]. There were no deviations from the initial protocol. The PRISMA [26] checklist allowed to increase the quality and transparency of the study, recorded in the PROSPERO (http://www.crd.york.ac.uk/PROSPERO accessed on 10 March 2021) database under number CRD42019139709.

### 2.2. Focused Question

What are the clinical outcomes of extra-short 4 mm dental implants?

### 2.3. Implants Definition

In the present SR, dental implants with a length of 4 mm were considered extra-short. In contrast, implants with a length >8 mm were considered longer [27].

### 2.4. Search Strategy

PubMed/MEDLINE, the Cochrane Central Register of Controlled Trials, Scopus, and Lilacs were used to search for articles that were published prior to August 2020, without any restrictions regarding date or language. A search of the gray literature using the Literature Report and Open Grey databases was also conducted. Finally, the study reference lists were evaluated (cross-referenced) to identify other potential studies for inclusion. MeSH terms, keywords, and other free terms related to “dental implant”, “short implant”, “short dental implants”, “posterior short dental implants”, “short dental implants maxilla”, and “short dental implants mandible” were used with Boolean operators (OR, AND) to combine searches. The search strategy included appropriate changes in the keywords and followed the syntactic rules of each database.

### 2.5. Eligibility Criteria and Study Selection Process

The inclusion criteria were based on the PICOST strategy [28]. The process of searching and selecting the studies was conducted in duplicate by two authors (D.A. and I.C.C). First, a careful evaluation of the titles and abstracts occurred, followed by a thorough assessment of the potential articles according to the eligibility criteria of this SR. The consensus of the two authors resolved possible disagreements. Only studies meeting the following criteria were included:

- Population: partially edentulous.
- Intervention: placement of extra-short 4 mm dental implants in the mandible
- Comparison: placement of long (>8 mm implants) in the mandible
- Outcomes: dental implant survival rate (primary outcome), marginal bone loss, biological complications (pain, exudate, peri-implant mucositis and peri-implantitis), and prosthetic complications (secondary outcome).
- Study design: RCTs and controlled clinical trials.
- Time: implants with follow-ups of 1–3 and 5 years

### 2.6. Data Synthesis

Data were extracted in duplicate by two authors (D.A. and I.C.C). The primary extracted data were authors, study design, number of participants, graft, outcomes, samples, follow-up, results, and conclusions.

### 2.7. Assessments of the Risk of Bias

The risk of bias analysis was performed by two reviewing authors (M.D.C.M. and I.C.C.). The Cochrane Collaboration’s tool for assessing risk of bias [29] was used for RCTs. Each study was analyzed in relation to six criteria: sequence generation, allocation concealment, blinding, incomplete outcome data, selective outcome reporting, and other sources of bias. Studies were classified as having low, medium, or high risk of bias when they met all, all but one, or all but two or more criteria, respectively.

### 2.8. Statistical Analysis

For binary outcomes (e.g., dental implant survival), the number of failures and the total number of placed implants were used to calculate the mean difference (MD) with a 95% confidence interval (CI). For continuous outcomes (e.g., MBL), the estimation of intervention effects was expressed as risk ratio (RR) using the mean and standard deviation values of bone loss in millimeters, with a CI of 95%.

The inverse variance method was used for the random-effect or fixed-effect models, depending on the heterogeneity between the studies. The heterogeneity was assessed using chi-square tests. Values ≤25% were validated as low heterogeneity, while values >25 and ≤50% were classified as moderate. Values ≥50% were classified as high heterogeneity. The use of the random-effect model was conducted when heterogeneity was found (*p* < 0.10). In contrast, the fixed-effect model was used in the case of low or medium heterogeneity. The statistical significance level of the effect of the meta-analysis was fixed at *p* < 0.05.

A funnel plot was drawn for the primary outcome variable (implant survival rate) to assess publication bias across studies. Studies outside the confidence interval area may indicate possible publication bias. The meta-analyses were conducted through software Review Manager (version 5.2.8, Cochrane Group, London, UK).

Because of the methodological differences, the prosthetic and biological complications analyses could not be conducted through meta-analysis. On the other hand, descriptive statistics were made.

### 2.9. Grading the Quality of Evidence

The quality of evidence (certainty in the estimates of effect) was investigated for each outcome using the Grading of Recommendations Assessment, Development and Evaluation (GRADE) approach [30]. For the analysis, issues such as risk of bias, inaccuracy, inconsistency, indirectness, and publication bias were identified.

## 3. Results

### 3.1. Literature Search

**Figure 1:**
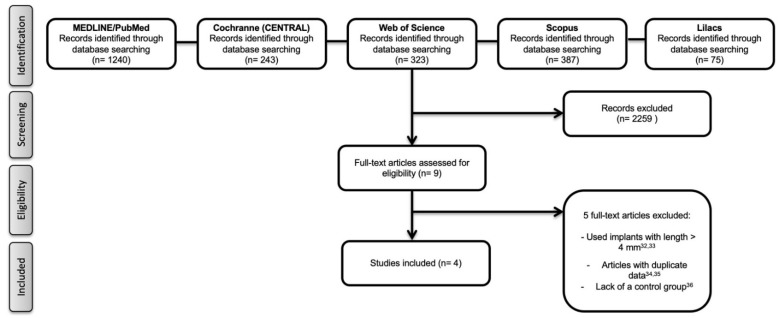
Flow diagram (PRISMA format) of the screening and selection process.

The initial search produced 1240 titles from MEDLINE/PubMed, 243 from the Cochrane Library, 323 from the Web of Science, 387 from the, and 75 from Lilacs. After the first evaluation (title and abstract assessment), 2259 articles were excluded. Of the nine potential articles, five studies [31,32,33,34,35] were excluded after careful reading because they did not meet the inclusion criteria [31,32], presented duplicate data [33,34], or lacked a control group [35]. Consequently, four studies [22,24,36,37], which were published between 2016 and 2018, were included in this SR. The search in the gray literature did not result in any further studies. The reasons for the exclusion of potential studies and the search and selection processes are presented in Figure 1.

### 3.2. Study Characteristics

One controlled clinical trial [36] and three RCTs [22,24,37] were included in the present SR. A total of 186 extra-short implants with a treated surface and 135 longer implants also with a treated surface were placed in the mandible of 301 research participants. All included studies used a late prosthetic loading protocol. All articles followed the implants up to 12 months, and no articles with longer follow-up were found. All implants were placed in the premolar and molar regions. In two studies [24,36], the implants were placed only in the mandible. Regarding the restorations, all studies used splinted implants with an internal prosthetic connection. Two studies [24,36] did not report information about smoking participants. The characteristics of the included studies are presented in Table 1.

### 3.3. Meta-Analysis and Quality of Evidence

The analysis showed high strength of evidence for the outcome implant survival rate. Regarding the MBL outcome, a moderate to weak strength of evidence was observed due to serious issues of imprecision and inconsistency (Table 2).

**Figure 2:**
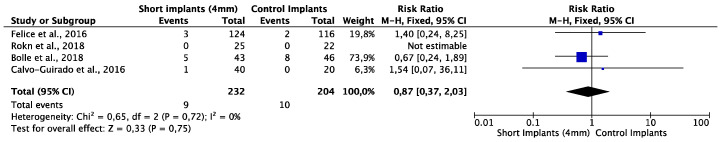
Funnel plot for the event of implant survival rate.

**Figure 3:**
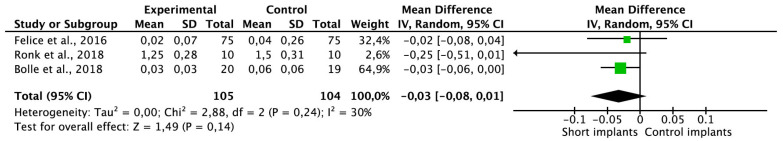
Funnel plot for the studies reporting the primary outcome “implant survival rate.”

The implant survival rate of the extra-short and longer implants after 12 months ranged from 88.4% [22] to 100% [22] (mean of 95.4% ± 4.97) and 82.7% [22] to 100% [24,36] (mean of 95% ± 8.29), respectively. The fixed-effect model was used to evaluate implant survival due to the lack of heterogeneity between the studies (*p* = 0.72; *I*^2^ = 0%). There was no statistically significant difference (*p* = 0.75) between extra-short and longer implants, with a RR of 0.87 (95% CI: 0.37 to 2.03) (Figure 2). The funnel plot demonstrated a fairly symmetrical distribution indicating a low risk of publication bias (Figure 3).

**Figure 4:**
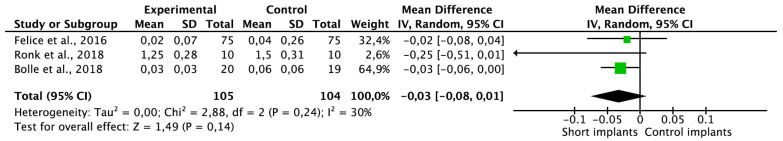
Forest plot for the event marginal bone loss immediately after implant placement.

**Figure 5:**
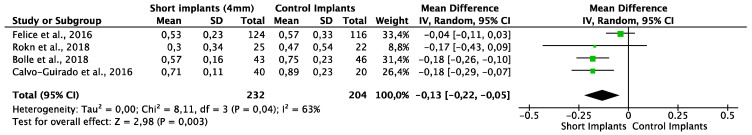
Forest plot for the event marginal bone loss after 12 months of prosthetic loading.

The mean MBL for extra-short and longer implants immediately after implant placement was 0.43 ± 0.7 and 0.53 ± 0.83, respectively. After 12 months, the mean MBL was 0.52 ± 0.17 mm and 0.67 ± 0.18 mm. When evaluated immediately after implant placement, the random-effect model was used to evaluate MBL due to the moderate heterogeneity between the studies (*p* = 0.24; *I*^2^ = 30%). There was no significant difference (*p* = 0.14) between groups, with a MD of −0.03 (95% CI: −0.08 to −0.01) (Figure 4). After 12 months of prosthetic loading, the random-effect model was used to evaluate MBL due to the heterogeneity between the studies (*p* = 0.04; *I*^2^ = 63%). The extra-short implants had a significantly (*p* = 0.003) lower MBL rate when compared to longer implants with a MD of −0.13 (95% CI: −0.22 to −0.05) (Figure 5).

### 3.4. Biological Complications

The biological complications most commonly reported in the included studies were pain, exudate, and mobility. Usually, these complications were associated with lost implants. The mean number of complications associated with extra-short implants was lower (3.4% ± 2.1) when compared to longer implants (11.6% ± 10.6).

### 3.5. Prosthetic Complications

Three studies [22,24,37] reported data about prosthetic complications. The number of prosthesis without complication of the extra-short and longer implants ranged from 95% [22] to 100% [24,37] and 84.7% [22] to 100% [24,37], respectively. A mean survival rate of 98.0% ± 2.4 was observed for extra-short implants and 95.5% ± 7.3 for longer implants. The most commonly reported prosthetic complication was screw loosening.

### 3.6. Assessments of the Risk of Bias

Two RCTs [22,24] were classified as low risk of bias, while two [36,37] others were classified as a moderate risk of bias (Table 3).

## 4. Discussion

### 4.1. Summary of Evidence

This SR is the first to analyze the clinical performance of extra-short 4 mm dental implants in the mandible. The use of extra-short implants in the posterior arches is a clinical strategy to avoid bone regeneration surgeries, reducing the surgical risk, time of treatment, cost, and, consequently, increasing patient acceptability. Thus, this study evaluated the null hypothesis that there were no differences in clinical outcomes between extra-short 4 mm dental implants and longer implants (>8 mm).

Some risk factors may be associated with the use of short implants such as lower primary stability [34], location of the implants in the cortical bone portion (lower cell viability), occlusal overload [38], and a poor crown–implant ratio [39]. However, recently, a SR [19] was published evaluating the effectiveness of 5 and 6 mm implants in a mean follow-up of five years. The authors concluded that 5 and 6 mm short implants are a viable treatment in long term for ridges exhibiting atrophy.

One study included [22] in the present SR showed lower survival rates (88.4%) of extra-short implants when compared to the other included studies. The authors attributed a greater number of failures to the inclusion of research participants with more aggressive bone atrophies (5.0 to 6.0 mm of bone above the mandibular canal or 4.0 to 5.0 mm below the maxillary sinus). Except for this study [21], the other works [22,34] observed survival rates above 92.2% (mean of 94.8% with the mean follow-up of 12 months). No study observed a significant difference between the implant survival rate of extra-short and longer implants.

Despite the high survival rate of extra-short implants found by this SR, these values are lower when compared to longer implants evaluated for a period of 120 months (mean of 96.5%), reported in other SR [4]. Both SRs evaluated implants with a regular diameter platform (≥3.75 mm).

The great majority of implant failures reported by the studies were early (absence of osseointegration). As all reports adopted late prosthetic loading, the failure reasons were probably related to the surgical technique (e.g., surgical instrumentation and primary stability) or to the healing process. Although the evidence for the reasons associated with the early implant failure is not robust, the implant surface roughness, bone quality, and the association of systemic diseases can be related factors [36].

Concerning MBL, there was a significant difference between the groups in favor of extra-short implants in the meta-analysis. The mean MBL for extra-short implants was 0.52 mm over a mean follow-up period of 12 months. According to Misch et al. [40], a successful implant (optimum health) should have a MBL < 2 mm from the initial surgery independent of follow-up time.

All the included studies [21,22,36,37] used splinted implants. Although there is no consensus in the literature, a recent SR [41] of finite element analyses observed that the highest concentration of biomechanical stress occurs in the cortical section (around the upper part of the implant). Thus, the study concludes that the diameter of the implants is more important than the length to minimize the peri-implant stress concentration. Although there is no consensus on the survival rate of splinted and nonsplinted implants, there is generally a greater tendency for nonsplinted implants to have a greater number of prosthetic complications [42].

In three studies [21,34,35], the authors started the prosthetic procedures after four months of healing and only one study [22] started after two months of healing. The study that waited for two months to start the prosthetic procedures got 100% of implant and prosthetic survival and lower MBL, so we did not find a correlation between the time for osseointegration and the implant and prosthetic survival.

Two studies [43,44] concluded that implant-supported fixed prostheses with crown–implant ratio > 2 do not correlate positively with MBL. However, the present SR did not observe a significantly higher MBL of extra-short implants when compared to long implants. There was no significant difference (*p* = 0.14) in MBL at the time of prosthetic loading between extra-short implants and long implants. However, after 12 months of function, extra-short implants showed a significantly (*p* = 0.003) lower MBL rate when compared to long implants. The fact that extra-short implants are splinted may explain the difference in MBL when compared to long implants. Another factor is the short follow-up time (12 months). There is a tendency for a higher occurrence of biological and mechanical failures related to short implants over three years of function [45].

Two studies [24,36] reported having included smokers’ participants. There is robust evidence of smoking patients tending to present higher MBL around dental implants when compared to nonsmokers [46,47,48]. Thus, the inclusion of smokers in the trials may cause a confounding factor in the interpretation of the results.

Biological complications in extra-short implants were observed by the included studies. Usually, these complications were associated with lost implants. In general, the studies found a higher number of prosthetic complications in the long implant group when compared to the extra-short implants. The most commonly reported complication was a loss of the prosthetic screw. Two other SRs that evaluated short (5–6 mm) [19] and longer implants [4] present this failure. Longer implants are usually rehabilitated through single crowns, explaining the higher rate of loosening of screws when compared to splinted implants.

### 4.2. Strengths and Limitations

The present SR demonstrates several strengths including a broad and unrestricted search process. Most studies are RCTs and presented a low risk of bias and relatively high quality in the strength of the outcomes (GRADE analysis). Another strong point is the homogeneity of the included studies. All studies installed the implants in premolar and molar areas with late prosthetic loading, with a follow-up of 12 months.

However, some limitations are present. First, a low number of studies with a long-term follow-up period are available in the literature (3–5 years of follow-up). Confounding factors as the inclusion of smoking participants, splinted implant-supported restorations, and type of material restoration (ceramic or resin) can make interpretation difficult and add bias in the results. Finally, all evaluated short implants were of a “tissue-level” platform with a smooth collar neck surface, and therefore this specific macrostructure could also impact the clinical outcomes. In this way, caution is required to interpret the present data.

### 4.3. Implications for Clinical Practice and Future Directions

The available evidence supports the use of extra-short 4 mm dental implants for the rehabilitation of partially edentulous posterior arches. However, the decision to rehabilitate the patient through extra-short implants or longer implants and bone regeneration should be evaluated independently for each patient.

The professionals’ clinical choice requires a careful evaluation of the occlusion, prosthetic area, bone atrophy extension, and patient preference. Researchers are encouraged to develop new longitudinal (five years or more) RCTs to assess the clinical performance of extra-short implants in the future.

## 5. Conclusions

In conclusion, after 12 months of follow-up, extra-short implants compared to the longer implants, did not show significant differences in survival rate, despite having presented greater marginal bone loss. These represent a viable clinical option with a low number of biological and prosthetic complications A higher number of RCTs with longer follow-up evaluating the clinical outcomes of extra-short 4 mm implants is necessary for the future. A higher number of RCTs with longer follow-up evaluating the clinical outcomes of extra-short 4 mm implants is necessary for the future.

## Figures and Tables

**Table 1:** Main characteristics of included studies.

Authors (Year)	Study Design No. of Participants	Follow-Up (Months)	Implant Location	No. of Implants Implant Brand Surface Type	Implant Size (Diameter × Length)	Loading Protocol	No. of Smokers	Primary Stability (Ncm or ISQ)	Implant Survival Rate (No. of Failures)	Marginal Bone Loss (Mean ± SD) (mm)	Prosthetic Complication (No. of Failures)	Biological Complications	Conclusions
Felice et al., 2016	RCT (parallel) 150	12	PM M	SI: 78 TwinKon Rough CI: 47 TwinKon Rough	SI: 4.0 × 4.0 LI: 4.0 × 8.5, 10, 11.5, 13	Delayed	SI: 20 LI: 12	>25 Ncm	SI: 96% (3) LI: 97.3% (2)	SI: 0.53 ± 0.23 LI: 0.57 ± 0.33	SI: 97.3% (2) LI: 97.3% (2)	SI: 4 LI: 2	This study indicated that 4.0 × 4.0 mm implants, one year after loading, achieved similar results to 8.5 × 4.0 mm long or longer implants in the presence of adequate bone volumes.
Calvo-Guitrado et al., 2016	CCT 60	12	PM M	SI: 40 Strauman Rough LI: 20 Strauman Rough	SI: 4.1 × 4.0 LI: 4.1 × 10	Delayed	NR	>25 Ncm	SI: 97.5% (1) LI: 100% (0)	SI: 0.71 ± 0.11 LI: 0.89 ± 0.23	SI: 100% (0) LI: 100% (0)	NR	Extra-short dental implants supporting single crowns or FDP are a feasible treatment option with radiographic and clinical success rates similar to longer implants for patients with compromised ridges
Rokn et al., 2018	RCT (parallel) 11	12	PM M	SI: 25 Strauman Rough LI: 22 Strauman Rough	SI: 4.1 × 4.0 LI: 4.1 × 8.0, 10	Delayed	NR	NR	SI: 100% (0) LI: 100% (0)	SI: 0.30 ± 0.34 LI: 0.47 ± 0.54	SI: 100% (0) LI: 100% (0)	SI: 0 LI: 8	This study showed that 4 mm dental implants and longer implants provided acceptable outcomes up to 1 year after loading
Bolle et al., 2018	RCT (parallel) 80	12	PM M	SI: 43 TwinKon Rough LI: 46 TwinKon Rough	SI: 4.0, 4.5 × 4.0 LI: 4.0 × 8.5, 10, 11.5, 13	Delayed	SI: 2 LI: 8	>25 Ncm	SI: 88.4% (5) LI: 82.7% (8)	SI: 0.57± 0.16 LI: 0.75 ± 0.23	SI: 95% (2) LI: 84.7% (6)	SI: 6 LI: 23	This study showed that, one year after loading, 4.0 mm long implants achieved similar results than longer implants and were affected by fewer complications.

RCT, randomized clinical trial; CCT, control clinical trial; n, number; M, molar; PM, premolar; Ncm, newtons per centimeter; ISQ, Implant stability quotient, SI, short implants; LI, long implants; NR, not reported; FDP, fixed dental prosthesis.

**Table 2:** Quality of evidence for the overall outcomes.

Outcomes	Anticipated Absolute Effects * (95% CI)		Relative Effect (95% CI)	No of Participants (Studies)	Certainty of the Evidence (GRADE)	Comments
	Risk with Extra-Short 4 mm Dental Implants	Risk with Long Implants				
Implant survival rate Follow up: mean 12 months	1.000 per 1.000	870 per 1.000 (370 to 1.000)	RR 0.87 (0.37 to 2.03)	436 (4 RCTs)	⨁⨁⨁⨁ HIGH	Nonsignificant effect
Marginal bone loss Follow up: mean 12 months	The mean marginal bone loss was 0.52 mm	MD 0.13 204 lower (0.29 lower to 0.07 lower)		436 (4 RCTs)	⨁⨁⨁◯ MODERATE ^a^^,^^b^	Significant effect

CI, confidence interval; RR, risk ratio; MD, mean difference; RCT, randomized clinical trial; * The risk in the intervention group (and its 95% confidence interval) is based on the assumed risk in the comparison group and the relative effect of the intervention (and its 95% CI). ^a^ The studies showed moderate heterogeneity; ^b^ The study by Felice et al. [38] presented a much larger sample size than other studies which increased the risk of publication bias.

**Table 3:** Assessments of the risk of bias of randomized clinical trials (Cochrane scale).

Authors (Year)	Adequate Sequence Generation	Allocation Concealment	Blinding	Incomplete Outcome Data Addressed	Selective Outcome Reporting	Free of Other Souces of Bias	Estimated Potential Risk of Bias
Felice et al., 2016	Yes	Unclear	Yes	Yes	Yes	Yes	Moderate risk
Calvo-Guirado et al., 2016	Yes	Unclear	Yes	Yes	Yes	Yes	Moderate Risk
Rokn et al., 2018	Yes	Yes	Yes	Yes	Yes	Yes	Low risk
Bolle et al., 2018	Yes	Yes	Yes	Yes	Yes	Yes	Low Risk

The studies that met all of the criteria were classified as having a low risk of bias, while those that did not meet a criterion were classified as having a moderate risk. When two or more criteria were not met, the studies were considered to have a high risk of bias.

## Data Availability

Not Applicable.
